# Mechanisms of Immune Evasion, Dissemination, and Persistence in *Leishmania amazonensis* Infection

**DOI:** 10.1111/pim.70044

**Published:** 2026-01-07

**Authors:** Arieli Bernardo Portugal, Renato Augusto DaMatta, Edézio Ferreira da Cunha Júnior, João Luiz Mendes Wanderley

**Affiliations:** ^1^ Laboratório de Imunoparasitologia, Instituto de Ciências Médicas, Universidade Federal do Rio de Janeiro Macaé Brazil; ^2^ Laboratório de Biologia Celular e Tecidual, Universidade Estadual Norte Fluminense (UENF), Campos dos Goytacazes Rio de Janeiro Brazil; ^3^ Instituto de Ciências Farmacêuticas, Universidade Federal do Rio de Janeiro Macaé Brazil

**Keywords:** dissemination, immune evasion, latency, *Leishmania amazonensis*, persistence, phosphatidylserine

## Abstract

*Leishmania amazonensis* is one of the etiological agents of cutaneous leishmaniasis in its localised form. Moreover, this parasite can cause more severe disease conditions, such as diffuse and disseminated cutaneous leishmaniasis. The development of more severe clinical manifestations is associated with the parasite's ability to establish a chronic infection and disseminate to cutaneous tissues distal to the site of primary infection. This ability is dependent on host immune factors but is also influenced by intrinsic factors of the parasite related to pathogenicity and evasion of the immune system. This review discusses immunological and parasitological factors associated with the latency and dissemination of *L*. *amazonensis* infection and the consequent development of severe forms of cutaneous disease.

## 1 Introduction

Leishmaniasis comprises a spectrum of clinical manifestations caused by protozoa belonging to the genus *Leishmania*. Notably, out of the 31 species that infect mammalian cells, 20 are described as pathogenic to humans [1]. Currently, cases of leishmaniasis are reported in 99 countries. Approximately 85% of cutaneous leishmaniasis (CL) cases are documented in Afghanistan, Algeria, Brazil, Colombia, Iran, Iraq, Pakistan, Peru and the Syrian Arab Republic. Conversely, about 68% of visceral leishmaniasis (VL) cases are concentrated in Brazil, Kenya, India, and Sudan. It is estimated that one billion people reside in areas at risk, and annually, approximately 30,000 new cases of VL and over 1 million new cases of CL are diagnosed [2].

Due to the variety of species related to the infection and the individual differences in the immune system of the vertebrate host, the clinical manifestations of leishmaniasis are diverse. The World Health Organization (WHO) classifies the clinical forms in humans as VL, CL, mucosal (ML), diffuse (DCL), disseminated (DL) and post‐kala‐azar dermal leishmaniasis (PKDL) [3].

Human infection occurs when hematophagous phlebotomine insects feed and regurgitate metacyclic promastigote forms of the parasite into the lesion they create. These forms infect cells of the phagocytic system and develop inside vacuoles into amastigote forms, which preferentially proliferate inside macrophages. However, amastigotes can be found and persist in various types of cells, such as dendritic cells, eosinophils, neutrophils, epithelial cells, myocytes and fibroblasts [4, 5, 6, 7, 8, 9]. During a new blood meal, phlebotomine sandflies collect amastigotes that differentiate into procyclic promastigotes in the posterior sandfly midgut. These forms undergo a process termed metacyclogenesis, culminating with the formation of metacyclic promastigotes that migrate to and accumulate in the sandfly anterior midgut, thus restarting the cycle [10, 11]. Considering the above, this study will address different aspects related to the chronicity and reactivation of *Leishmania amazonensis* infection and the relationship of these mechanisms with the development of severe clinical manifestations of CL caused by this species.

### 1.1 L. Amazonensis

*L. amazonensis* is one of the species associated with cases of CL in the Americas. While not the primary etiological agent, it is endemic and capable of causing various types of clinical manifestations and severe complications of the disease [12]. Cases of *L*. *amazonensis* infections are primarily distributed in the Amazon Rainforest region, spanning to countries such as Bolivia, Brazil, Colombia, Costa Rica, Ecuador, French Guiana, Panama, Peru, Suriname and Venezuela. In Brazil, infections caused by *L*. *amazonensis* exhibit a wide geographical distribution, occurring in all country regions [13].

Human infection is accidental, as the primary mammalian hosts in this parasite's natural ecosystem are wild rodents, such as species of the genus *Proechimys* [14]. Currently, there are records of this parasite infecting non‐human primates, felines, and dogs [15, 16, 17, 18, 19]. Infections in domestic animals, especially dogs, indicate the urbanisation of the parasite and an increased risk of transmission to humans [20]. The main vector is the sandfly species *Lutzomyia flaviscutellata*. However, there are reports of infections in regions where this insect species does not occur, indicating the adaptation of the species to other vectors [19].

This species is primarily associated with cases of CL, but there are reports of VL, ML, DCL and cutaneous relapses [21, 22, 23, 24]. The exact mechanisms that induce these different clinical presentations are still unknown. Therefore, due to the multiplicity of clinical manifestations, *L*. *amazonensis* is an excellent subject for studying the understanding of pathogenesis and immune evasion in chronic infection.

*L. amazonensis* is responsible for a variety of clinical manifestations, including DL and DCL, also known as anergic diffuse cutaneous leishmaniasis, which are severe and rare forms of CL that can cause permanent sequelae. Clinical manifestations occur several months or years after the initial lesion. Understanding the pathogenesis of these clinical forms remains a significant challenge and achieving a therapeutic cure is difficult [25, 26]. In DCL, parasites grow uncontrollably, causing diffuse cutaneous lesions without the characteristic ulceration observed in CL [27]. In DL, dozens of papulonodular lesions appear throughout the body, preferably on the chest and limbs. In this clinical form, the parasite spreads hematogenously, presenting as non‐ulcerated nodular lesions with chronic evolution and intense dermal involvement. These lesions, except mucous membranes and scalp, can cover the entire skin, with numerous parasites and recurrent episodes. Patients exhibit a low response to treatment attempts and chemotherapy resistance. Additionally, a negative result in the Montenegro skin test is quite common, complicating diagnosis and indicating a lack of cellular immune response [28].

## 2 Immune Response to *L. amazonensis* Infection

The clinical manifestations of leishmaniasis display a varied array of immune responses, leading to diverse manifestations and complications. Infections by *L*. *amazonensis* exhibit an even more complex scenario, as the dichotomy of immune responses observed in infections with other *Leishmania* species cannot be seen in cases involving *L*. *amazonensis* [29, 30]. Silveira's study noted that 49% of patients with CL caused by *L*. *amazonensis* were unable to respond to the Montenegro test despite harbouring the parasite in their bodies. This might be related to a negative regulation of adaptive cellular response induced by the infection [31].

Variations in immune responses found in *L*. *amazonensis* infections are mainly related to differences between strains [32, 33]. The direction of the response is defined in the first interaction with the host. It involves a combination of cytokines, chemokines, Toll‐like receptors (TLRs), and the presence of dendritic cells in this initial interaction. This infection typically induces an inefficient and limited T‐cell response [34]. The heterogeneity of the immune response in patients infected with *L*. *amazonensis* can be exemplified by the exaggerated T helper 1 (Th1) cell response in ML lesions and the absence or low activation of T cells in DCL lesions [35]. Patients infected with *L*. *amazonensis* exhibiting different clinical manifestations present an antibody repertoire that recognises different antigens, demonstrating the polyclonality of the humoral response in infected patients [36]. In infections with *Leishmania major*, susceptibility to the infection is related to the immune response skewing towards a Th2 cell profile, with the production of anti‐inflammatory cytokines such as interleukin (IL) 4, IL‐10, IL‐13 and transforming growth factor‐beta (TGF‐β), as well as the presence of M2 macrophages, eosinophilia, increased polyamine production, decreased nitric oxide (NO) production and increased production of neutralising IgA and IgG1 antibodies [37]. Conversely, resistance to infection is associated with a Th1 profile, characterised by high production of IL‐12, interferon‐gamma (IFN‐γ) and tumour necrosis factor‐alpha (TNF‐α), and the presence of M1 macrophages with high NO production [35, 38]. In animal models, after *L. major* infection, BALB/c mice progressively develop lesions, serving as models for infection susceptibility, whereas C57BL/6 mice infected with *L. major* heal spontaneously and are used as models to study infection resistance [39].

However, in *L*. *amazonensis* infection, the dichotomous pattern of Th1/Th2 response and resistance/susceptibility does not occur. The inflammatory profile formed during the infection is heterogeneous, and lesions occur independently of the genetic background of the experimental mouse model [40, 41]. In animal models, even the CH3/He mouse strain, considered resistant to *Leishmania* infection, exhibits a mixed inflammatory response (Santos‐Pereira 2019).

Comparing *L*. *amazonensis* infection with *L. major* , it is observed that in the initial phase of *L*. *amazonensis* infection there is low production of inflammatory cytokines such as IFN‐γ, interleukin 1β (IL‐1β), macrophage inflammatory protein‐α (MIP‐1α) and monocyte chemoattractant protein‐1 (MCP‐1) and of chemokine receptors CCR1, CCR2 and CCR5. These inflammatory markers increase production/expression throughout the infection, especially after treatment. Conversely, the production of anti‐inflammatory cytokines such as IL‐4 and IL‐10 is high at the beginning of the infection. It tends to decrease as the infection progresses and after a pharmacological cure. It is important to note that IL‐10 production is primarily responsible for inhibiting inflammatory cytokines and chemokines in the initial phase of infection [34]

A known mechanism is the ability of *L*. *amazonensis* to modulate the expression of arginase 1, ornithine decarboxylase, prostaglandin‐E2 and TGF‐β in the vertebrate host. Arginase 1 is an essential enzyme for polyamine synthesis, induced by the presence of anti‐inflammatory mediators such as TGF‐β and PGF2, and its expression reduces the expression of inducible nitric oxide synthase (iNOS) [42]. Recently, it was demonstrated that *L*. *amazonensis*'s arginase 1 can also modulate differentiation and virulence factors, such as reducing the expression of LPG, PPG and GP63 [43]. The amount of PGE2 induced in cutaneous infections does not significantly vary but differs from samples of healthy individuals, indicating its role in *Leishmania* infection (Table 1) [48]. The microenvironment entirely influences PGE2 activity; in the presence of pro‐inflammatory mediators such as TNF‐α, it induces the production of IL‐12. However, in combination with TGF‐β it blocks the production of IFN‐γ and TNF‐α [52, 53, 54].

**TABLE 1:** Principal cells and factors associated with *Leishmania amazonensis* dissemination.

Cell/factor	Change/observation	Effect on infection	References
Macrophages	Reduced integrin function	Decreased adhesion; increased migration	[44]
Dendritic cells	Altered migration and maturation	Impaired antigen presentation	[45, 46]
CCR4/CCR7	Downregulation of CCR4; upregulation of CCR7	Modified trafficking to lymph nodes	[45, 47]
Arginase 1, ODC	Elevated enzyme activity	Increased polyamine synthesis and parasite growth	[48]
PGE2, TGF‐β	High production levels	Suppression of proinflammatory cytokines	[48]
Cytotoxic molecules	Decreased perforin, granzymes, granulysin	Reduced parasite killing	[49]
T cells	Predominant CD8+ subset, reduced CD4+	Impaired cytotoxic control	[50]
B cells	Increased IgG, mainly IgG4	Anti‐inflammatory, non‐protective response	[51]

## 3 Key Pathogenicity Factors Associated With *L. amazonensis* Infection

Several pathogenicity factors operate to facilitate infection and enable the survival and dissemination of the parasite. Hence, these molecules can be present in the amastigote and promastigote forms. Although *L*. *amazonensis* possesses various pathogenicity factors like other species, there are some specific differences. The primary pathogenicity factors of the parasite include complexes of glycoconjugates modified by phosphoglycans (PG) linked to lipids, proteins, or free forms. Among these are lipophosphoglycans (LPG), glycosylphosphatidylinositol (GPI), proteophosphoglycans (PPGs) and glycoprotein 63 (GP63) [55]

### 3.1 LPG

LPG is expressed in promastigote forms [56] and is related to the early stages of infection, being essential for binding and entry into macrophages through complement receptors CR3 and lectin‐type receptors [57]. Additionally, it protects the parasite from microbicidal mechanisms such as complement‐mediated lysis, oxygen radicals and hydrolases [58, 59]. LPG exhibits significant structural polymorphism among *Leishmania* species and strains, with alterations in side chains and glycosylation levels, enabling the differential regulation of NO induction, pro‐inflammatory cytokines production and macrophage activation [60]. This structural diversity partly explains why LPG from species related to CL induces higher NO production and pro‐inflammatory cytokines than LPG derived from species related to VL [40, 61].

The structural diversity of LPG encompasses different strains of *L*. *amazonensis*, but its modifications are not related to distinct clinical manifestations or differences in the vertebrate host [40]. *L. amazonensis* metacyclic forms, purified by negative selection using a monoclonal antibody against procyclic LPG that agglutinates these parasite forms, are capable of resisting complement activation and producing more severe in vitro and in vivo infections in C57BL/6 mice, indicating that LPG is important for the establishment of infection, as observed in other *Leishmania* species [62]. In addition, *L*. *amazonensis* LPG is associated with the induction of neutrophil extracellular traps (NETs) and the activation of protein kinase R (PKR), leading to parasite death and control of the parasitic load at the infection site. In murine macrophage infections, it can stimulate NO and pro‐inflammatory cytokine production via TLRs 4, similar to *Leishmania mexicana* [63].

*L. mexicana* is phylogenetically grouped in the same complex as *L*. *amazonensis* [64] and its LPG is recognised by TLR2 and TLR4, inducing pro‐inflammatory cytokine production in human macrophages. Patients with DCL caused by *L. mexicana* exhibit a reduced quantity of natural killer (NK) cells in lesions and blood compared to patients with CL. LPG can bind to TLR2 of NK cells. In DCL patients, TLR2 expression is reduced compared to CL patients. In CL patients, TLR2 expression tends to be high at the beginning of the infection but decreases as the infection progresses. NK cells from DCL patients produce less IFN‐γ and TNF‐α compared to CL patients. This interaction contributes to parasite survival and dissemination by negatively modulating TLR2 expression in NK cells. This reduced recognition of LPG inhibits IFN‐γ and TNF‐α production [65].

### 3.2 PPGs and Free Glycolipids

Free GPI and protein‐conjugated GPIs are present in all *Leishmania* species and are essential for host–parasite interactions. They form a macromolecular barrier that protects promastigote forms from microbicidal processes such as complement‐mediated lysis, oxygen radicals and hydrolases found in the insect vector and/or vertebrate host [66]

GP63 is the primary metalloproteinase found in *Leishmania* membranes and plays a crucial role in macrophage infection. Its expression occurs in promastigotes and amastigotes and is anchored to the plasma membrane through a GPI anchor. In promastigotes, GP63 is vital for the interaction between the promastigote and macrophage and the parasite's survival within macrophage phagolysosomes [67]. The work of de Araújo Soares et al. [58] links the parasite's pathogenicity to the quantity of expressed metalloproteases, finding that the loss of pathogenicity during in vitro passages is directly associated with reduced GP63 expression [58]. Although more pathogenic strains have lower GP63 expression, they also exhibit higher proteolytic activity, likely contributing to their increased resistance to the complement system [68]. PPGs are glycoproteins that can be membrane‐anchored or secreted. Both promastigotes and amastigotes produce PPGs, but there are differences in the quantity and type of PPGs produced among different species [69, 70]. *L. mexicana* amastigotes secrete a significant amount of PPGs into the parasitophorous vacuoles, a characteristic feature also observed in *L*. *amazonensis* infection [71]. This vacuole enlargement aids the invasion and survival of the parasite within macrophages, providing nutrition and dilution of parasiticidal substances [72]. Interactions involving these glycoproteins are associated with drug resistance [73]. PPGs may also contribute to infection dissemination, as they can induce the production of anaphylatoxins C3a and possibly C5a, attracting monocytes and neutrophils to the infection site, which participate in parasite dissemination [74].

## 4 Factors Related to the Dissemination of *L. amazonensis*

The dissemination of the parasite is associated with different forms of leishmaniasis, including LD, LCD, LMC and PKDL. One of the primary reasons for these manifestations is the host's immune system's inability to eliminate the parasite. Specific cellular immunity is crucial for controlling leishmanial infections. However, in these patients, there is a failure in this response, which can be congenital, acquired through infection or caused by some other unknown event [25]. This phenotype plays a central role in the permissiveness generated by the immune system, allowing the parasite to replicate uncontrollably and spread throughout the body, leading to chronicity and the severity of the infection.

While immune surveillance is essential, there is evidence that intrinsic factors of the *Leishmania* species may play an equally or more significant role in developing severe forms of CL. In cases of *Leishmania*‐HIV coinfection, primarily documented in Africa and Asia, the main complication in patients infected with species causing cutaneous infection, such as *L. major*, is the dissemination of the parasite to internal organs such as the liver and spleen [75]. Additionally, even in immunocompromised individuals, clinical manifestations vary in dermotropic leishmaniasis [76]. Thus, intrinsic characteristics of *L*. *amazonensis* should be considered regarding the pathogenesis of severe CL.

Upon inoculation into the vertebrate host, parasites are rapidly phagocytosed, primarily by resident macrophages, monocytes and neutrophils [77]. A cutaneous lesion can develop at the site; however, healing can occur spontaneously or with chemotherapy. In other clinical forms, parasite migration and dissemination occur while the initial lesion is still active or after clinical cure [12].

For the dissemination process to occur, migration of the parasitized cells from the site of inoculation to adjacent epithelial tissues is necessary. Cell migration is a complex process involving chemokines and adhesion molecules such as selectins and integrins. Figueira et al. [78] described that *L*. *amazonensis* infection leads to integrin dysfunction and reduced adhesion capacity of leukocytes to connective tissues. The ability of cells to migrate or remain at the site is essential for determining lesion characteristics and distribution [79]. After *L*. *amazonensis* infection, macrophages showed reduced integrin function, leading to decreased macrophage adhesion, a critical mechanism for migrating infected cells to other tissues (Table 1) [44].

Species associated with visceralization, such as *Leishmania infantum*, induce increased migration of chemokine‐induced dendritic cells. Similarly, *L*. *amazonensis* strains isolated from patients with LCD and LMC also induce increased migration of cells compared to cells induced by LC‐derived *Leishmania* (Table 1) [45]. However, Hermida et al. [79] and de Menezes et al. [80] showed that migration of dendritic cells and macrophages parasitized with *L*. *amazonensis* was inhibited, suggesting that the persistence of these cells could aid in the infection's latency process and favour the late appearance of ulcers, a characteristic feature of LCD. Thus, integrin‐mediated cell migration following *Leishmania* infection could be an important mechanism for parasite dissemination and latency and modulating the host's immune response. This modulation occurs differently within the same species, requiring an analysis of strain characteristics.

G protein‐coupled chemokine receptors are related to integrin function. CCR4 is one such receptor mainly expressed in macrophages, dendritic cells, NK cells and T cells, predominantly in the Th2 phenotype. It is related to directing lymphocytes specifically to the site of inflammation. A study by Pinheiro et al. [47] showed that *L*. *amazonensis* infection induced negative regulation of CCR4 and phagocyte adhesion loss (Table 1) [47]. However, in Coelho et al.'s work [81], it was demonstrated that cells producing high levels of IFN‐γ express less CCR4, while cells producing low IFN‐γ express more CCR4 [81]. Infections with *L*. *infantum* and *L*. *amazonensis* LCD increase the expression of CCR7, a molecule related to cell migration to draining lymph nodes; there is also an increase in CCR7 expression in species that increase migration (Table 1) [45]. Therefore, the dynamic regulation of chemokine receptor expression by infection may be relevant in defining susceptibility and dissemination.

The factors mentioned above are possible mechanisms modulated by the infection to enable the parasite's dissemination to other tissues. When comparing patients with LCD and other manifestations, differences in some immune system processes can be found. Arginase 1, ornithine decarboxylase, PGE2 and TGF‐β are elevated in patients with LCD, systemically and at the infection site, contributing to parasite proliferation, disease chronicity and the formation of characteristic lesions. The systemic presence of PGE2 in CL, together with TNF‐α and IL‐12, induces immune system activation capable of controlling the infection. At the same time, LCD patients present high levels of PGE2 and TGF‐β, suppressing the immune system and allowing parasite proliferation and dissemination (Table 1) [48].

Lesions derived from LCD patients have lower levels of cytotoxic effector molecules, such as granulysin, perforin and granzymes A and B, compared to other forms of CL, which may contribute to the immune system's inability to control the infection (Table 1) [49]. Additionally, CD4+ and CD8+ T cells are found in smaller quantities in LCD lesions than in other manifestations, with CD8+ T cells being the predominant T cells at the inflammatory site (Table 1) [50]. However, they are not considered essential for dissemination, as they are not directly associated with cytotoxic control of infected macrophages. B cells are more frequent in LCD lesions than in LC patients and healthy individuals. LCD patients have higher levels of all IgG isotypes, with IgG4 accounting for 40% of all immunoglobulins. IgG4 does not protect the host, as it does not bind to complement and does not activate FcγRs; moreover, it contributes to the disease's anti‐inflammatory and refractory condition (Table 1) [51].

The disease's dissemination and outcome differ from other species in cases of borderline disseminated cutaneous leishmaniasis (LCBD) related to *L*. *amazonensis*. In this variation of the clinical manifestation, the disease manifests chronically, with the appearance of one or multiple lesions and not always involving mucosal tissues, differentiating it from LCD [82]. The first lesions appear after 6 months of healing the initial LC lesions, with a maximum of six metastatic lesions and a duration of 1–2 years. The dermis shows many vacuolated and heavily parasitized macrophages surrounded by lymphocytes and plasma cells. Parasite dissemination occurs through the lymphatic route due to the parasite's presence in lymph nodes. This parasite dissemination does not occur in LC and LCD forms, and the reason is still uncertain. However, a possible explanation is the change in the profile of Th2 TCD4+ cells to the Th1 profile. The significant presence of lymphocytes in LCBD lesions is a distinctive feature of LCD. It is crucial for the success of pharmacological treatment, as patients are considered cured after 6 months of treatment [49].

Lesions caused by *L*. *amazonensis* differ from other species, even when responsible for the same clinical manifestations. In LC, histopathological analysis shows that when *L*. *amazonensis* infection occurs, the inflammatory infiltrate is granulomatous, mainly composed of densely vascularized macrophages, unlike infections with *Leishmania braziliensis*, which have few macrophages and low parasite load, more lymphocytes, and plasma cells, characterising an epithelioid granuloma. In LCD lesions, similarly to LC lesions, the main histopathological characteristics are the intense infiltration of macrophages, many amastigotes, but with low frequencies of lymphocytes and plasma cells, giving the infiltrate a macrophagic granuloma appearance [49].

BALB/c nude mice can be used as possible study models for LCD, as both have reduced lymphocyte quantities. When these animals are infected with *L*. *amazonensis* in the paw and compared with BALB/c mice, it is observed that the lesions in nude mice are smaller, with decreased CD11b cell quantities and an increase in the granulocyte population. In nude mice infected with *L*. *amazonensis*, an increase in proteins related to oxidative stress, such as Oligopeptidase B (OPB) and tryparedoxin, also occurs [83]

## 5 Cells Involved in the Dissemination of *L. amazonensis* Infection

One of the hypotheses proposed is that parasite dissemination occurs through infected cells themselves, such as neutrophils, macrophages and dendritic cells (Table 1), as parasitized macrophages can be found in draining lymph nodes or in the blood in different clinical forms [84]. Neutrophils are the first cells to arrive at the infection site and, thus, interact with the parasites [85]. They are recruited from the blood and migrate to the infection site through the endothelium. The lifespan of circulating neutrophils is relatively short, and their role in infection development is still uncertain. These cells interact with parasites and other immune cells, secrete cytokines and chemokines that can control parasite or promote infection progression depending on the *Leishmania* species and host genetics [86]. Neutrophils have various mechanisms to eliminate the parasite, such as reactive oxygen species (ROS) production, phagocytosis, degranulation and extracellular trap release [87]. In infections with *L. major* and *L. mexicana*, live parasites can be found inside neutrophils, and their survival may be related to dissemination. This mechanism is called ‘Trojan horse’ because after the phagocytosis of promastigotes, neutrophils have their programmed death delayed, allowing interaction with monocytes that migrate to the infection site later and then, phagocytose apoptotic neutrophils. As the recognition and phagocytosis of apoptotic cells occur tolerogenically, with TGFβ production by phagocytes, parasites internalised within neutrophils can establish an intracellular infection in monocytes/macrophage [88]. This mechanism operates for other intracellular parasitic infections such as *Trypanosoma cruzi* [89]. Shortly after neutrophil infection with *L*. *amazonensis* promastigotes, many neutrophils die through NETosis, resulting in parasite entrapment and degradation [90]. The surviving neutrophils undergo apoptosis that is delayed by the parasite infection [91]. Approximately half of the promastigote population dies after neutrophil interaction [92].

Infection of neutrophils by amastigotes is also possible, but little is known about this interaction. In Carlsen et al.'s work, after infection with amastigotes, neutrophils had reduced TNF‐α production and increased IL‐10 production. Amastigote infection can also induce apoptosis in neutrophils [92]. Neutrophils can suppress T cell activity due to high levels of arginase 1 found in CD15+ neutrophils or an increase in programmed death‐1 protein (PD‐1) expression. In the chronicity of the disease in patients with VL and DCL, an increase in programmed death‐ligand 1 (PD‐L1) and PD‐1 occurs. In animal models, blocking the PD‐1 receptor and its ligand leads to decreased parasite loads and increased IFN‐γ production [86].

Dendritic cells specialise in antigen presentation and receive signals from the innate immune response, which are converted into appropriate stimuli for T cells. The two main populations residing in the skin are dermal dendritic cells and Langerhans cells. Langerhans cells' primary function is to phagocytose and process particles, but they are weak stimulators of T cells. When stimulated, such as by the endocytosis of a parasite, they reduce phagocytic capacity and increase the expression of major histocompatibility complex (MHC), co‐stimulatory molecules and adhesion molecules; all of them are important for activation of naive T cells. In *Leishmania* infection, Langerhans cells have fewer intracellular parasites in relation to macrophages, showing that they can restrict parasite replication [93]. Dermal dendritic cells are essential for initiating cellular responses [94]. Langerhans cells can transport ingested parasites from the infection site to lymph nodes. This migration occurs between 24 and 48 h after infection and is related to Langerhans cell that differentiate into dendritic cells capable of stimulating T cells [93].

*L*. *amazonensis* can infect dendritic cells, but these infected cells do not fully differentiate and express less CD1a and CD80. Moreover, infected dendritic cells have reduced antigen presentation capabilities, since they reduce MHC class II surface expression. Infection induces lower secretion of IL‐6, IL‐10 and IL‐12 during maturation. These modulations occur with both live and heat‐killed parasites, indicating that factors related to dendritic cell changes, such as glycolipids or lipids, are intrinsic to the parasite. The reduction in CD1a and CD80 expression shows that *L*. *amazonensis* infection modulates dendritic cell development and hampers the induction of T cell immune responses against the parasite, a crucial feature in *L*. *amazonensis* infection for parasite survival and dissemination [46, 95]. Dendritic cells also play a significant role in parasite dissemination by being one of the principal cells related to the transport of parasites from the inoculation site to lymph nodes, which are sites of parasitic proliferation in disseminated infections due to the presence of a large number of macrophages [96]. The main cells and factors associated with *L*. *amazonensis* dissemination are summarised on Table 1.

## 6 Latency in *L. amazonensis* Infection

In endemic areas of leishmaniasis, there is difficulty in the differential diagnosis between relapse or new infection, resulting in the misinterpretation of the actual infection scenario, parasite distribution and identification of latent infections [97]. The latent phase of infection is characterised by a period during which the parasite remains viable in the organism after a clinical cure. This period can be asymptomatic, and the disease may recur months later. It is a common mechanism in various infections, such as viruses, bacteria and parasites [97]. Parasite latency is described in human clinical manifestations in visceral and tegumentary forms [98, 99]. After clinical cure and parasite latency, lesions can recur in severe clinical manifestations such as MCL, VL, LD, LCD, HIV coinfection and PKDL [36, 98, 100, 101]. The reasons why the parasite remains in the organism after spontaneous cure and/or successful therapy are still unknown. However, viable parasites persist in the lymphatic system and dermis, mainly in dendritic cells and fibroblasts, and can also infect adipocytes [102]. The parasites live in these cells in low quantities and benefit from the failure of these cells' microbicidal mechanisms' activation [103, 104].

During spontaneous healing of LC lesions, parasites remain inactive in cells of the reticuloendothelial system. Although spontaneous healing of the lesion is associated with the development of the cellular immune response against the pathogen, this response is not always sustained, and over time, more severe clinical manifestations may appear [105, 106]

The production of IFN‐γ and IL‐10 is essential for modulating parasite latency, as mice deficient in IL‐10 can have a sterile cure, while excessive IL‐10 presence results in progressive, non‐healing lesions with a high parasite load. IFN‐γ is also present during the parasite's latency period, but due to IL‐10, cells become less responsive to IFN‐γ. During the parasite's latency phase, CD4+ T cells play an important role due to their ability to produce IFN‐γ and IL‐10. Parasite proliferation and lesion recurrence are related to the imbalance and impairment of CD4+ T cells and alterations in the production of these cytokines [103, 107].

During the chronic phase of *L*. *amazonensis* infection, atypical B cells are predominant with high antibody production, mainly IgG4. This is coupled with the absence of T‐cell cytotoxicity and regulatory macrophages incapable of eliminating the parasite [51].

Fibroblasts are essential in *L*. *amazonensis* infection and its clinical manifestations due to the large quantity of these cells at the infection site, and studies have shown them to be susceptible cells to infections [5, 108]. Primary cultures of skin fibroblasts have mannose receptors on their surface, which participate in parasite entry similarly to what has been described for macrophages. Infection with viable amastigotes controls receptor expression on these cells. The control of latent infection in these cells occurs due to the activation of an inflammatory response and the inefficient parasitic proliferation in fibroblasts. With parasite death, there is a decrease in mannose receptor expression and decreased permissiveness of these cells for new infections [109]. Cavalcante‐Costa [5] demonstrated that mouse embryonic fibroblasts are susceptible to infection and parasite entry occurs actively, with temporary plasma membrane permeabilization, calcium signalling lysosome recruitment/exocytosis and lysosome‐mediated endocytosis, very similar to *T. cruzi* [5].

One way the parasite can subvert the host's immune response and survive chronically in immune cells is by the modulation of extracellular signal‐regulated kinase MAPK (ERK1/2) in infected dendritic cells and macrophages. The parasite is capable of modulating ERK1/2 activation through phosphorylation. ERK1/2 activation inhibits the expression of surface molecules CD40 and IL‐12p40 production, altering dendritic cell maturation and contributing to the non‐polarised T cell phenotype characteristic of non‐healing *L*. *amazonensis* infection lesions. Another contribution of ERK1/2 activation is to increase the survival of the parasitized cell. Blocking ERK1/2 leads to increased ROS and reactive nitrogen species (NOS) production, demonstrating an important role in infection chronicity [110, 111].

## 7 Phosphatidylserine (PS) as a Pathogenic Factor for Dissemination and Latency of *L. amazonensis*

One of the pathogenic mechanisms employed by *L*. *amazonensis* is termed apoptotic mimicry. Apoptotic mimicry involves the use of apoptotic molecular features as a mechanism to interact, regulate and infect host cells [112]. Apoptotic mimicry also occurs in other organisms, such as viruses, protozoa, and tumour cells [112, 113, 114, 115, 116, 117]. *L. amazonensis* infective forms make use of PS exposure in this context [118]. PS exposure serves two main functions: (1) inducing parasite endocytosis and (2) modulating cytokine and chemokine production as well as immune polarisation, leading to an anti‐inflammatory profile in phagocytes. PS exposure occurs in both amastigotes and promastigotes but in different ways. In promastigotes, apoptotic mimicry presents itself in a non‐classical form [113]. In this case, PS is exposed in dead metacyclic forms that exhibit apoptotic characteristics such as oligonucleosomal DNA degradation and chromatin condensation. The presence of these forms is crucial for the establishment of the infection, as they induce the downregulation of macrophage activation, allowing the establishment of infection by viable metacyclics [119]. In amastigote forms, PS exposure is constitutive and not accompanied by other characteristics of cell death. Amastigote forms take advantage of this feature, allowing infection and proliferation within macrophages [120].

Recognition of PS exposed by *L*. *amazonensis* induces the production of anti‐inflammatory cytokines such as IL‐10 and TGFβ [118]. As discussed earlier, IL‐10 production is related to inhibiting anti‐inflammatory cytokines and chemokines in the initial stages of the lesion, essential for the establishment of the infection. Its presence is crucial for infection latency, suggesting that PS exposure possibly influences infection establishment and latency.

In promastigotes, PS exposure decreases as they are maintained through consecutive in vitro passages [121], possibly related to the loss of pathogenicity observed in other studies under similar conditions [122]. Concomitant with reduced PS exposure, promastigotes maintained in vitro for extended periods induce higher IFN‐γ production and lower IL‐10 production in macrophages, resulting in smaller lesions in mice. It is associated with modifications in the lipophosphoglycan molecule [123], decreased capacity for differentiation into amastigotes [122] and possibly reduced PS exposure [121].

As discussed earlier, PS recognition on amastigote surfaces is crucial for macrophage infection. However, other cells are involved in latency and subsequent parasite dissemination in manifestations such as LCD and LD. Dendritic cell infection by the parasite appears not to be partially PS‐dependent. When PS is blocked during interaction with the parasite, dendritic cells present more antigens and are more competent in activating specific CD4+ T lymphocytes against the parasite [95]. Thus, PS on amastigote surfaces may contribute to immune system suppression, a requirement for parasite dissemination. Little is known about the involvement of PS recognition in the infection of other cells involved in latent infection and parasite dissemination, such as fibroblasts and neutrophils.

TGF‐β is the main cytokine stimulated by PS recognition on amastigote surfaces [118] and can modulate the inflammatory microenvironment, providing a suitable site for parasite proliferation. This cytokine is found at higher levels in LCD patients. *L*. *amazonensis* isolates obtained from LCD patients exhibit higher PS exposure than those from LC patients. In LCD patients, PS exposure of parasites correlates directly with the number of lesions and the disease duration [48]. PS exposure is suggested to correlate with cutaneous dissemination and parasite resistance to treatment. An essential characteristic of LCD is resistance to pharmacological treatment. As described earlier, one of the main factors related to antimony resistance is increased expression of ABC transporter proteins. Campos‐Salinas et al. [124] demonstrated that negative modulation of LABCG2, a transporter of the ABC family, reduces PS exposure in promastigotes. Thus, polymorphisms in this transporter family might contribute to drug resistance by eliminating drugs from inside the parasite and modulating PS exposure and, consequently, the inflammatory and immune response.

One of the changes found in LCD patients is the low expression of TLR2/4 in NK cells compared to cells derived from LC patients. Parker et al. [125] demonstrated that PS prevents TLR4/2 complex formation in peripheral blood mononuclear cells, causing these receptors to lose activity. Pro‐inflammatory cytokines were inhibited, as was IL‐10 production [125]. However, it is important to note that a balance in IL‐10 production is necessary for proliferation and latency to prevent complete parasite elimination and achieve parasitological cure.

Spleen‐derived apoptotic cells induce increased CCR4 expression [126]. PS exposure leads to low IFN‐γ expression, which might result in high CCR4 expression. This relationship could be related to the migration of phagocytes from the *Leishmania* inoculation site and the development of systemic/adaptive disease. Low CCR4 expression is associated with phagocyte adhesion loss and increased IFN‐γ expression. These events might be related to a possible parasite attempt to escape inflammatory microenvironments.

## 8 Conclusion

We have observed that *L*. *amazonensis* can modulate the host's immune system in a way that allows infection to be established and possibly disseminated to various tissues within the vertebrate host. This modulation is primarily based on suppressing the inflammatory and immune response. Several immune system cells are crucial for this process, and the key cytokine involved is IL‐10. Although the mechanisms related to latency and relapse are not as well elucidated as those of dissemination, immune system evasion is necessary for the parasite to persist within the organism. However, it is essential to emphasise the need for further studies on the influence of intrinsic pathogenicity factors such as surface protein and lipid glycoconjugates, as well as PS exposure, in the development of LCD, LD, and other disseminated forms of cutaneous infection.
